# The societal economic impact of vision impairment in adults 40 years and above: findings from the National Eye Survey of Trinidad and Tobago

**DOI:** 10.1038/s41433-023-02860-x

**Published:** 2023-12-08

**Authors:** T. Braithwaite, H. Bailey, D. Bartholomew, V. Maharaj, A. Fraser, F. Deomansingh, S. S. Ramsewak, V. Tripathi, S. Sharma, D. Singh, S. S. Ramsewak, R. R. A. Bourne, A. Gray

**Affiliations:** 1 School of Life Course and Population Sciences, King’s College London, London, UK; 2 The Medical Eye Unit, Guy’s and St Thomas’ NHS Foundation Trust, London, UK; 3 Department of Economics, The University of The West Indies, St. Augustine Campus, Trinidad and Tobago; 4 Ophthalmology Department, Port of Spain General Hospital, Trinidad, Trinidad and Tobago; 5 Richmond University Medical Center, Staten Island, NY USA; 6 Department of Optometry, The University of the West Indies, St Augustine Campus, Trinidad and Tobago; 7 Today’s Optical Ltd, Trinidad, Trinidad and Tobago; 8 Department of Mathematics and Statistics, The University of the West Indies, St Augustine Campus, Trinidad and Tobago; 9 Standard Trust Capital Partners Ltd, St Augustine, Trinidad, Trinidad and Tobago; 10 Caribbean Eye Institute, Valsayn, Trinidad, Trinidad and Tobago; 11 Faculty of Medical Science, The University of the West Indies, St Augustine, Trinidad and Tobago; 12 The Vision and Eye Research Institute, Anglia Ruskin University, Cambridge, UK; 13 Ophthalmology Department, Addenbrooke’s Hospital NHS Foundation Trust, Cambridge, UK; 14 Health Economics Research Centre, Nuffield Department of Population Health, University of Oxford, New Richards Building, Old Road Campus, Headington, Oxford, UK

**Keywords:** Health care economics, Risk factors, Vision disorders, Outcomes research, Epidemiology

## Abstract

**Background:**

Understanding and mitigating the societal economic impact of vision impairment (VI) is important for achieving the Sustainable Development Goals.

**Aim:**

To estimate the prevalent societal economic impact of presenting VI in Trinidad and Tobago using bottom-up cost and utilisation data from the 2014 National Eye Survey of Trinidad and Tobago.

**Methods:**

We took a societal perspective to combine comprehensive, individual-level cost and utilisation data, with population-based prevalence estimates for VI, and additional data from a contemporaneous national eyecare system survey. We included direct (medical and non-medical) and indirect (productivity loss) costs, and intangible losses in total cost estimates, presented in 2014 Trinidad & Tobago (TT) dollars and UK sterling equivalent. We considered but excluded transfer payments and dead weight losses. Sensitivity analyses explored impact on total cost of parameter uncertainty and assumptions.

**Results:**

Individual utilisation and cost data were available for 65.5% (*n* = 2792/4263) and 59.0% (*n* = 2516/4263) eligible participants aged ≥40 years, respectively. Participant mean age was 58.4(SD 11.8, range 40–103) years, 56.3% were female. We estimated total societal cost of VI in 2014 at UK£365,650,241 (TT$3,842,324,655), equivalent to £675 per capita (population ≥40 years). Loss of wellbeing accounted for 73.3%. Excluding this, the economic cost was UK£97,547,222 (TT$1,025,045,399), of which indirect costs accounted for 70.5%, followed by direct medical costs (17.9%), and direct non-medical costs (11.6%).

**Conclusion:**

This study provides a comprehensive estimate of the economic impact of vision loss in a Caribbean country, and highlights the extent to which affected individuals and their families bear the societal economic cost of vision impairment.

Understanding and mitigating the societal economic impacts of vision impairment (VI) will be critically important for achieving the Sustainable Development Goals (SDGs) [1–3]. The global cost of VI was estimated at US$3 trillion in 2010, and projected to rise 20% by 2020 [4]. More recently, annual, global productivity losses from VI (<6/18) were estimated in the 15–64 year old population in 2018 at $410.7 billion purchasing power parity, equivalent to 0.3% of global Gross Domestic Product [5, 6]. Despite the importance of this problem, there has been little population-based observational research to support detailed estimation of the economic impact of VI in different countries [6]. Considered from a societal perspective, economic cost encompasses not only the direct costs resulting from eye care services, treatments and non-medical costs, and indirect costs resulting from lost income (productivity losses), but also wellbeing impacts on affected individuals and their carers. The Disability Adjusted Life Year (DALY) was developed to capture such intangible effects of disease [7]. Globally, there were an estimated 22.6million DALYs associated with vision impairment in 2019, accounting for 0.88% of DALYs from all causes [8].

Cost-of-illness studies estimate the economic burden associated with a disease or health state, through describing, itemising, valuing and summing the associated costs [9]. Their usefulness to policy makers has been debated since their inception over 50 years ago, with arguments advanced by proponents [10, 11], and critics [12]. Arguably, cost-of-illness studies provide the most valuable insight when designed as descriptive, bottom-up studies that capture, “the true cost to society”, “envisage the different subjects who bear the costs”, and explain sources of cost variability, to help direct further investment or research effectively [13]. Through highlighting the impact of disease on both the population and the economy, they can also provide a basis for prioritising research funding across different disease areas [14]. Every country has a unique population and political, social welfare, employment and healthcare systems, which influence the economic impact of VI. Previous systematic reviews of cost of vision loss studies reveal very few based on primary observational research, with no prior data from national eye surveys, and frequent omission of the productivity loss associated with informal care [5, 6, 15]. Study heterogeneity has precluded meta-analyses [6, 15]. Additionally, there are no data on the economic impact of vision loss in the Caribbean [6, 15].

To address this, we included an economic questionnaire in the National Eye Survey of Trinidad and Tobago (NESTT, 2014), the most comprehensive population-based eye survey undertaken in the Caribbean region for over two decades [16, 17], and in a contemporaneous national eyecare health system survey [18]. Herein we estimate the total societal economic cost in 2014 resulting from presenting VI involving the better-seeing eye, adhering to cost categories outlined by Cost of Vision Loss Consensus Guidelines (2010) [19]. Additional descriptive data are provided to give more nuanced insight into the economic and wellbeing impacts of VI in Trinidad and Tobago in 2014, and access to low vision support.

## Methods

Full details on the methodology and main results from the NESTT (2014) are provided elsewhere [17, 18, 20]. In brief, we sampled 9913 eligible people aged 5 years and above, residing in 3556 households (95.9% coverage) within 120 clusters, using multi-stage, random cluster sampling with probability-proportional-to-size methods, including 4263 people aged 40 years and above. In 3589 (84.2%) responders, we measured uniocular presenting distance, and binocular presenting near visual acuity outside the household, according to a standardised measurement protocol [17]. The demographics of NESTT participants aged 40 years and above were similar to the 2011 Census data for the same age group [21], with mean age of 57.1 (sd 11.8) years, 54.3% female (versus 50.6% in Census), most of South Asian (43.7% versus 35.4% in Census) or African (40.0% versus 34.2% in Census) ancestry with the remainder of predominantly mixed race (15.2% versus 22.8% in Census), and 4.4% (versus 4.6% in Census) resided in Tobago [16]. Adults (≥40 years) were invited for comprehensive clinic-based assessment, including administration of multiple questionnaires, administered using the Epi Info software package (version 3.5.4, Centers for Disease Control and Prevention, Atlanta, GA, USA).

### Socioeconomic variables

We administered a socioeconomic questionnaire (Supplementary Table 1), adapted for inclusion in this study [17, 21, 22]. In the clinical assessment, we recorded spectacle and contact lens ownership, surgical and laser history. We recorded prescription topical ophthalmic medication use over the past 3 months. We asked participants with best-corrected visual acuity worse than 6/18 about access to low vision services and use of low vision aids.

### Statistical methods

We performed statistical analyses using standard statistical software (StataCorp.2013.Stata Statistical Software:Release 13.1.College Station,TX:StataCorp LP). We report crude estimates for means or proportions in participants. We report ‘adjusted’ estimates for means or proportions in the population-representative sample. We adjusted crude estimates using STATA’s ‘svy’ command suite, to account for multi-level survey design (by island and cluster), with weighting for selection probability, and variable response rate by cluster (weight = 1/response rate). Post-stratification adjustment used the national Census (2011) for the non-institutional population (stratified by 15 municipalities, gender and 5-year age categories). We applied finite population corrections to the first and second sampling stages, including the total number of enumeration districts, and households, by island.

We used multi-level mixed effects logistic regression models (STATA ‘melogit’) to account for multi-level survey design by island and cluster, adjusted for age and sex, and study design, to explore odds of: presenting VI (<6/12) by employment group or educational attainment or literacy, utilising any eye care services, having health insurance, and of using the public sector exclusively for eye care, by vision category.

### Cases of vision impairment

We estimated total cases in each vision category [23], by applying adjusted prevalence estimates to the 2014 mid-year population aged 40 years and above (541,894 people) [24].

### Classification of economic impact

We estimated direct and indirect costs in each category (See Table 1) [19]. We took a societal perspective to estimate all prevalent costs associated with VI in adults (≥40 years) in Trinidad and Tobago in 2014, regardless of who incurred them. We included costs to individuals, family and friends providing informal care, the Government, health care system, and employers (in the form of productivity losses) [25].

**Table 1:** Definitions and estimation of cost categories in Trinidad & Tobago in 2014.

Cost category	Definition	How data were collected and costs estimated in this study and explanatory notes
Direct costs		
a) Medical Direct costs	Costs included the resources used to treat an eye disease, including eye care services provided by ophthalmologists, optometrists, health centres, general practitioners and the emergency department, eye surgery, medication for eye care, laser therapy and other ophthalmic interventions.	EYE CARE SERVICE UTILISATION: We asked survey participants how many times in the past year they visited each group of eye care provider, and if more than once, how many times they visited. From this prevalence of utilization, and the mean number of episodes of utilization amongst those reporting any, both of which we adjusted, we estimated episodes of eye care service utilization per annum for each group of providers in 2014 (Supplementary Table 2). EYE CARE SERVICE COSTS: We multiplied these by the mean unit cost for each eye care service (Supplementary Table 3). We estimated eye care service unit costs in a contemporaneous study on the eye care system in Trinidad and Tobago in 2014 [17]. In this study, we contacted all registered eye care providers in Trinidad and Tobago by post or email or telephone, inviting them to complete questionnaires. These included the public and private sector tariffs for their outpatient, inpatient and emergency eye services and eye treatments. Provider groups included ophthalmologists, optometrists, public hospital eye department administrators, health centres, and public sector GPs. Further cost information sources included personal communication with officers within the Ministry of Health. Unit costs were not available for public hospital day case and overnight admission, and public sector laser, and the cost of these was therefore assumed to be the same as private sector costs. TOPICAL OPHTHALMIC MEDICATION USE AND COST: In the national survey, we ascertained the number and name of prescription topical ophthalmic medications used in each eye in the past 3 months, and asked about compliance (Supplementary Table 4). We assumed these applied to the past 12 months for the person. We obtained private sector unit costs for 13 commonly used topical ophthalmic medications in 2014, from three private companies operating in Trinidad and Tobago in 2014, who were invited to submit competitive tender to supply drugs to the NESTT study. We averaged these to produce a mean unit price per drug. We identified public sector unit costs from the Government Chronic Disease Assistance Program (CDAP) price list for the same year. We used public sector unit costs if participants reported free topical ophthalmic medications, private sector unit costs if participants reported paying for all topical ophthalmic medication, and an average unit cost if participants reported using a mixture of free and paid prescriptions. LASER: NESTT participants were asked to report whether they had received any laser therapy (and what type) in the private sector in the past 12 months, or ever (and what type) (Supplementary Table 5). In the health system survey we asked public and providers to report laser procedure volumes for 12 m in 2013-2014, and fees. OTHER EYE TREATMENTS: NESTT participants were asked to detail all surgical or other medical treatments for the eyes (Supplementary Table 6). In the health system survey we asked each of the 5 regional ophthalmology departments, and private ophthalmology clinic responders, to report on surgical services offered and costs.
b) Non-medical direct costs	Costs included refractive correction, low vision aids, and transportation to attend eye care services.	REFRACTIVE CORRECTION: The health system survey determined that spectacles and contact lenses were exclusively available in the private sector in 2014, and in addition, basic reading spectacles could be purchased over-the-counter in some supermarkets. The cost of basic distance and near spectacles, and of bifocal, trifocal and varifocal spectacles and contact lenses was determined in the eye care system study, from 48 registered optometrists who responded to the questionnaire (Supplementary Table 3) [17]. We asked survey participants whether they had purchased spectacles or contact lenses in the past 12 months as an out of pocket expenditure (Supplementary Table 7). We used the adjusted prevalence to estimate the number of pairs of spectacles purchased in 2014. We assumed that 70% of spectacles purchased were basic distance or near spectacles, and 30% were bi, tri or varifocal spectacles to estimate total cost (Supplementary Table 8). LOW VISION SUPPORT: We invited those with low vision (best-corrected visual acuity in the better seeing eye worse than 6/18) to complete an additional questionnaire, in which they were asked whether they had received a low vision assessment in the past year, and whether they owned any low vision aids (Supplementary Table 9). The list of low vision aids included devices to assist the individual in their personal, home and work environments, and was developed in consultation with the Low Vision Clinic at the University of the West Indies in St Augustine, Trinidad, and the Blind Welfare Association in Port of Spain, Trinidad, in 2013. The adjusted prevalence of low vision was used to estimate the number of cases in Trinidad and Tobago in 2014, and the crude proportion using each type of low vision aid was used to estimate low vision aid purchases in 2014. The unit cost of low vision assessment was ascertained directly from optometrists in the eye care system study [17]. The unit price of individual low vision aids in Trinidad and Tobago in 2014 was not determined. A literature review identified a study on the cost of low vision aids in four European countries in 2004 [46], which has been used in other cost of vision loss studies in the USA and UK [47, 48]. Additional unit costs were obtained from the Royal National Institute for the Blind UK online shop [49]. These costs were adjusted to 2014 values. TRAVEL FOR EYE CARE: Survey participants who reported attending an eye care service in the past 12 months were asked what their usual mode of transportation was (e.g. private car, taxi, water taxi, bus), and the crude proportion was applied to the total estimated episodes of eye care to estimate the number of return journeys of each type (Supplementary Table 10). Mean unit costs associated with return journeys using different modes of transportation were obtained from 450 outpatients attending eye clinics in the five regional hospital ophthalmology departments [17].
Indirect costs		
a) Productivity loss associated with VI	The value of lost labour output caused by reduced economic productivity resulting from VI in the affected individual. We excluded the cost of allowances (benefits, financial support for income, residence) and consideration of the time that visually impaired people spend in prevention activities or self-help groups.	We invited survey participants to specify the category into which their household monthly income fell but individual income was not ascertained, as the latter was felt to be too sensitive a question for inclusion in the NESTT. It was therefore not possible to directly determine the mean reduction in income associated with different categories of VI. Instead, we used a simple human capital approach to estimate productivity loss. We defined the employment rate (ER) as the percentage of the population of working age in this study (40 years to 64 years, inclusive) who reported being employed over the past 12 months. We estimated the adjusted ER for each vision category (Supplementary Table 11). To estimate lost productivity, we assumed that in the absence of VI, individuals would have been employed at the same rate as the average person aged 40 to 64 years in Trinidad and Tobago in 2014. We calculated the employment ‘gap’ as 1 minus the ER in that vision category divided by the overall ER in the population aged 40 to 64 years. We calculated productivity loss for individuals in each vision category as the product of the employment gap, median annual income in 2014, and overall ER [36, 50]. For the base cost case, we used a median annual income for all occupations in 2014 of TT$54,000 [51]. In sensitivity analysis we explored two alternatives from the same Central Statistics Office data, namely the average annual income for all occupations in 2014 of TT$66,960, and the average annual income for elementary occupations of TT$40,704. We multiplied the productivity loss for individuals in each vision category by the estimated number of VI cases in the 2014 population aged 40 to 64 years, using adjusted prevalence estimates for this age group, and summed to obtain total productivity loss. To calculate the productivity loss associated with part-time work, we used the same analysis approach, assuming a 50% reduction in working hours (Supplementary Table 12). We took this approach to be conservative, because it did not account for the possibility that those with VI might experience slower promotion or restricted choice over employment type and associated lower earning potential than those with normal vision [52]
Productivity loss associated with sick leave	The value of lost labour output caused by reduced economic productivity resulting from VI in the affected individual.	We asked participants who reported employment how many sick days they had taken in the past 12 months in total, and specifically whether they had taken any days off to attend healthcare services for their eyes or vision (Supplementary Table 13). We also asked them to specify the total value of any lost earnings over that period. Workers who are absent on account of illness for a protracted period are likely to be replaced at some point. This period, the ‘friction period’ (e.g. 90 days), can be used to make an adjustment to the productivity loss to avoid overestimating the actual loss [53]. We did not apply a friction period adjustment in this study because the number of days of sickness taken on account of incapacity from eye disease or VI was not directly ascertained, and was anticipated to be few days on average.
Informal care	The value of lost labour output caused by reduced economic productivity resulting from the care of an individual with VI	We asked participants if any friends or family members provided them with informal care on account of their eyes or vision state in the past month, and if so, we asked them to specify how many hours (Supplementary Table 14). To estimate the value of productivity loss associated with informal care, we used the opportunity cost method [54]. Specifically, we used the proportion reporting need for informal care to estimate the number of people, multiplied by the mean hours of informal care per person needing any, and by the hourly wage rate for an individual in an elementary occupation, of TT$21.20. The latter was calculated from the mean annual income of an elementary occupation in 2014 of TT$40,704, assuming 40 working hours per week and paid annual vacation of 4 weeks per year [51].
Transfer payments	Payments between economic agents, for example, social welfare payments made for distributional purposes rather than as payment for goods or services.	We asked participants about their employment status over the past 12 months, to estimate the prevalence of individuals having formal ‘disabled’ status (Supplementary Table 16). We assumed that these individuals were in receipt of disability allowance if they were aged between 40 and 64 years. The means-tested disability allowance was $1500 per month in 2014 and was available up to the age of 64 years, after which it was replaced by the senior citizens pension of TT$3000 per month. We reported the budgetary impact associated with this social welfare disability payment in the total cost estimate. There was no specific carers allowance in Trinidad and Tobago in 2014. We also reported the budget cost of Government-funded programs providing services for the blind, identified from documents in the public domain, in the total cost estimate, but did not include fiscal flows resulting from reduced income tax revenue. We excluded this category from final cost estimates.
Dead weight losses	The excess allocative inefficiency on society associated with administering transfer payments and raising additional taxation revenue [15]. Examples of dead-weight loss include welfare payments resulting in reduced labour force participation, and taxation levels disincentivising people from working.	Dead-weight losses are challenging to estimate reliably, and we did not estimate them in this study, in line with most other cost studies.
Intangible effects		
Intangible effects	The suffering associated with a condition or disease may greatly extend beyond financial costs. Intangible effects are defined as the loss of wellbeing experienced by affected individuals.	It is challenging to quantify loss of wellbeing, and even more challenging (and controversial) to assign a monetary value to the loss. Previous cost of illness, and cost of vision loss studies specifically, have seldom assigned a monetary value to loss of welfare (Koberlein et al., 2013), but current Consensus Guidelines recommend this for cost of VI studies (Frick et al., 2010). In this analysis we adopt the common approach of estimating Disability Adjusted Life Years (DALY, Murray, 1994) associated with vision impairment, and present total cost estimates both with and without intangible effects.
	Disability weights: A number between 0 (perfect health) and 1.0 (a health state as bad as death)	Disability weights were introduced in the 1990s to give a new population health measure, the disability adjusted life year (DALY). Disability weights are obtained from ordinal measurement of preferences (paired health state comparisons). Advanced modelling is required to transform these data into weights. At least eight studies have used a variety of methodological approaches to estimate disability weights associated with vision impairment, with blindness disability weights varying from 0.17 to 0.60 [27].
	Disability adjusted life years (DALYs): DALYs aim to capture a societal assessment of the burden of a disease resulting from premature mortality and the non-fatal consequences of disease, in terms of lost welfare, subjective wellbeing and quality of life.	DALYs facilitate explicit comparison of health outcomes for health sector planning and evaluation, and greater consistency in resource allocation decisions. DALYs differ from quality adjusted life years (QALYs), which measure individual preferences for time spent in different health states (Supplementary Table 15).

### Definition and estimation of intangible effects

To estimate DALYs experienced by the population aged 40 years and above in 2014, we adopted the approach taken by the World Health Organization and Institute of Health Metrics for the Global Burden of Disease Study to estimate loss of wellbeing. In this, prevalent years lived with disability (YLD) are estimated, and years of life lost (YLL) assigned a value of zero to reflect an assumption that VI is not directly associated with premature mortality [26]. To calculate prevalent YLD, we multiplied prevalent cases by the disability weight, with no discounting for time or unequal age weights. After consideration of all available weights [27], we chose the latest WHO disability weights, of 0.047 for near VI (with normal distance vision), 0.005 for mild distance VI, 0.089 for moderate distance VI, 0.314 for severe distance VI, and 0.338 for blindness [26], but explored alternatives at the high [7] and low [28] extremes in sensitivity analysis. We reported impact of VI on quality-adjusted life years previously [29].

Trinidad and Tobago is a high-income non-OECD (Organisation for Economic Co-operation and Development) country, with no published in-country estimation of the value of a statistical life (VSL). Taking the life expectancy at birth to be 70.52 [30], and using a VSL derived for Trinidad and Tobago in 2015, of US$3,035,000, we estimated the value of one lost year of wellbeing in 2015 to be US$43,037, without discounting [31]. We applied the World Bank GDP deflator to yield a VSL in 2014 of $42,483 [32], equivalent to TT$272,316.

### Sensitivity analysis

In sensitivity analysis we varied multiple parameters within their 95% confidence interval to explore impact on total cost of various assumptions. Where primary data were not available, or available for only a subgroup of participants, we varied the parameter by +/−50%.

### Allocation of costs

Some costs are easier to attribute than others. For direct costs, we applied public or private sector unit costs depending on the participant’s stated preferred provider; if participants reported using both sectors for eye care, we applied an average of the public and private sector unit costs. We assumed that the Government, via the regional health authorities, bore the cost of public sector eye care and treatments made available on the Chronic Disease Assistance Program (CDAP). We assumed that individuals bore the cost of transportation, all refractive correction, and private eye care services and treatments. We assumed insured individuals had a 30% co-payment, based on survey responses.

### Conversion of unit costs

We inflated unit costs in this analysis, where necessary, to 2014 values, using the World Bank Gross Domestic Product deflator [32], which takes into account fluctuating exchange rates and different purchasing power of different currencies and inflation rates. We then converted into TT$ using The World Bank Official Exchange Rate (LCU per US$ period average) in 2014, which was TT$1 = UK£0.0952 [33].

### Ethics approval

We obtained Ethics Committee approval from the Ministry of Health of the Government of the Republic of Trinidad and Tobago, the University of the West Indies (Trinidad), and Anglia Ruskin University (UK). The study adhered to the tenets of the Declaration of Helsinki. All participants provided written informed consent.

## Results

We estimated eyecare service utilisation (Supplementary Table 2) and indirect costs from the NESTT medical and ophthalmic questionnaires (2792 (65.5%) completed), and socioeconomic questionnaire (*n* = 2,516 (59.0%) completed). Participants included 72.5% (161/222) of all participants with mild VI, 73.5% (158/215) with moderate or severe VI and 41.9% (13/31) of those who were blind in the better-seeing eye. We obtained direct costs in the contemporaneous eyecare system survey (Supplementary Table 3) [18].

### Total economic cost of vision impairment and eye care

The total societal cost of VI amongst adults 40 years and above in Trinidad and Tobago in 2014 was TT$3,842,324,655 (UK£365,650,241), with loss of wellbeing accounting for 73.3% of total cost (Table 2). Excluding this, the economic cost was TT$1,025,045,399, of which indirect costs accounted for 70.5%, followed by direct medical costs (17.9%) and direct non-medical costs (11.6%). These equated to a one-year per capita cost to every member of the adult population (≥40 years) of £674.76 including lost wellbeing, and £180.01 excluding lost wellbeing, of which £32.19 were direct medical costs, £20.96 were direct non-medical costs, and £126.86 were indirect costs. Supplementary Tables 2–15 present detailed estimates underlying these summary costs, and Table 16 details excluded transfer payments.

**Table 2:** Cost of vision loss in Trinidad and Tobago in 2014 in adults ≥40 years, subdivided into cost categories (TT$s 2014 (TT$1 = UK£0.0952 ()).

Cost group cost item (sector, if applicable)	Total COST, TT$	Data presented in supplement, table number
1.a. Direct Medical		
Optometrist (private) appointments	10,487,167	2, 3
Ophthalmologist (both)	53,339,693	2, 3
GP (private)	691,531	2, 3
Health centre (public)	3,148,044	2, 3
Emergency department (both)	1,017,023	2, 3
Overnight admission (both)	14,203,343	2, 3
topical ophthalmic medication bottles prescribed (both)	66,612,229	4
Laser therapy to anterior segment for posterior capsule opacity or glaucoma (34.8%) or retina (65.2%)	7,963,698	5
Day case cataract surgery (both)	25,841,007	2, 3
Other ophthalmic treatments/surgery	0	6
Direct medical subtotal	183,303,734	
% excluding intangible effects	17.9	
1.b. Direct non-medical		
Transportation to access eye care	11,394,239	10
Spectacles or contact lenses (private)	70,391,870	7, 8
Low vision aids and assessment (private)	37,576,201	9
Direct non-medical subtotal	119,362,310	
excluding intangible effects (%)	11.6	
2. Indirect		
Productivity loss from VI	630,859,320	11
Productivity loss - part time work	80,276,228	12
Productivity loss - sick leave	6,211,776	13
Productivity loss - informal care	5,032,032	14
Indirect subtotal	722,379,355	
excluding intangible effects (%)	70.5	
TOTAL COST	1,025,045,399	
excluding intangible effects (%)	100.0	
including intangible effects (%)	26.7	
3. Intangible effects		
Prevalence DALYs in 2014	2,817,279,256	15
including intangible effects (%)	73.3	
TOTAL COST	3,842,324,655	
including intangible effects (%)	100	

### Estimation of cases with vision impairment in 2014

We estimated 64,431 (95% CI 54,623–74,077) cases of distance VI amongst adults 40 years and above in 2014 [16]. Of these, 86.1% (95% CI 82.9 to 88.8), equivalent to 55,481 (95% CI 53,401–57,221) cases, were potentially avoidable. In addition, there were an estimated 120,842 (95% CI 112,715–128,971) cases of avoidable near VI resulting from uncorrected presbyopia.

### Sensitivity analyses

**Fig. 1:**
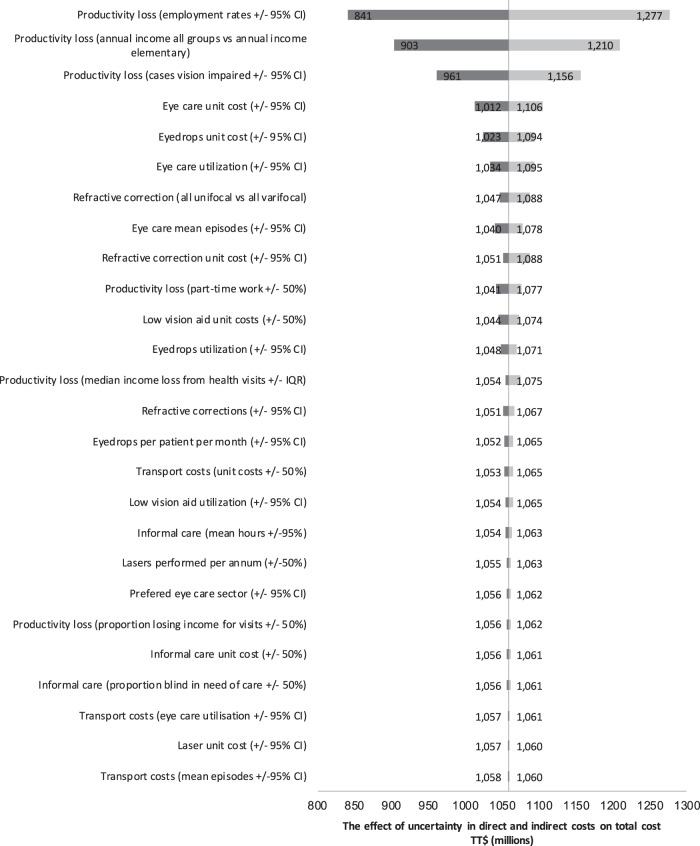
Tornado chart illustrating the effect of uncertainty in direct and indirect costs on total cost (in TT$ millions). In this special form of bar chart, each bar represents a cost variable/source of uncertainty, ordered with those making the greatest contribution to uncertainty in the total cost estimate at the top, and those making the smallest contribution to uncertainty in the total cost estimate at the bottom.

A Tornado chart (Fig. 1) illustrates the outcome of a 1-way deterministic sensitivity analysis exploring impact of parameter uncertainty on the base cost estimate, excluding loss of wellbeing. Applying alternative disability weights from the Global Burden of Disease (GBD) Study (2013) resulted in substantial reduction in cost associated with lost wellbeing, to just 3485 DALYs, equivalent to TT$941,661,752 [26]. In contrast, applying the original GBD Study disability weights increased the estimate to 12,259 DALYs, equivalent to TT$3,338,445,299 [7].

### Differential economic impacts by vision level

Whilst we could not ascertain individual participant income to permit direct monetary estimation of productivity loss, data from 2663 responders revealed an association between greater levels of presenting VI and lower household income (Supplementary Table 17). The employment rate varied by category of presenting vision, from 73.2% (95% CI 70.9–75.4) in those with both normal distance and near vision (*n* = 1110/1561), to 41.4% (95% CI 30.6–52.2) in those with moderate or severe vision impairment [MSVI] (*n* = 28/69), with no employment amongst blind individuals (*n* = 0/5) (Supplementary Table 11). This yielded individual productivity losses ranging from TT$2,367 in those with near VI only, to TT$36,869 in those who were blind, whilst those with normal distance and near vision had a productivity gain of TT$2639 compared to the population median.

The three most frequent occupational categories in Trinidad and Tobago in 2014 using the Information Commissioner’s Office classification were professionals (37.6%), elementary occupations (20.6%), and service workers or market sales people (18.2%), whether VI or normally sighted (Supplementary Table 18). In a multivariable model, the odds of having presenting VI (<6/12) were significantly higher amongst people employed in the service industry (OR 2.7, 95% CI 1.3–5.4) and people employed in elementary jobs (OR 2.9, 95% CI 1.5–5.8), as compared to professionals (*p* = 0.011). Considering other variables potentially associated with occupational opportunity, an active email address was reported by 31.4% (56) with normal vision, 9.5% [17] with mild VI, 4.4% [8] with MSVI, and no one with blindness. Access to a family car was reported by 75.5% (1712) with normal vision, 64.3% (128) with mild VI, 61.9% (122) with MSVI, and 50.0% [14] who were blind.

Individuals with vision impairment had significantly lower educational attainment than those with normal vision (See Supplementary Table 19). In a multivariable model, the odds of presenting with vision <6/12 in the better seeing eye were significantly lower amongst those who had completed secondary school (OR 0.43, 95% CI 0.31–0.59), post-secondary (OR 0.44, 95% CI 0.25–0.77) or university (OR 0.30, 95% CI 0.15–0.62), as compared to those who had completed only primary school (*p* < 0.0001). Illiteracy was reported by 0.74%(11/1483) of people with normal distance vision, compared to over 2.5% of those with mild VI (5/199), MSVI (5/196) and blindness (1/30). In a multivariable model the odds of presenting with vision <6/12 were significantly reduced in those who were literate (OR 0.26, 95% CI 0.12–0.58), compared to those who were illiterate (*p* < 0.001).

### Eye care access by vision level

Eye care service utilisation in the past 12 months varied from around 30% in those with normal vision, mild VI and MSVI, to 18% in those with near VI and 7% in those who were blind (Supplementary Table 20). A multivariable model revealed that use of eye services was significantly more likely in women (OR 1.36, 95% CI 1.13–1.64), and amongst people older than 60 years, whereas those with near VI (OR 0.49, 95% CI 0.39–0.61), or blindness (OR 0.11, 95% CI 0.01–0.85), were significantly less likely to report utilising any eye care.

Health insurance coverage also varied significantly by vision level, ranging from 24.6% (95% CI 21.8–27.6) in those with normal distance and near vision, to 0% in blind participants (*p* < 0.001) (Supplementary Table 20). Median (IQR) out-of-pocket-expenditure (OOPE) on eye care ranged from TT$100 (100–150) for optometry sight tests, reported by 16.0% (402) in the past 12 months, to TT$10,000 (1600–13000) for day case surgery, reported by 0.7% [18] (Supplementary Table 21). Any OOPE on eye care services in the past 12 months was reported by 25.3% (95% CI 22.9–27.8) of those with normal vision, and by no one who was blind (Supplementary Table 21). OOPE on topical ophthalmic medications were incurred by 19.3% (95% CI 9.9–34.1) of blind people, compared to only 4.7% (95% CI 3.7–6.0) amongst those with normal vision.

## Discussion

This study provides the first estimate of the societal cost of presenting VI in Trinidad and Tobago. We estimate a prevalent cost of TT$3.8 billion (UK£365.7 million) to the general adult population 40 years and above in the year 2014, with loss of wellbeing accounting for 73.3%, followed by indirect costs, then direct medical and non-medical costs. Affected individuals and their families bore the major share of total cost (97.6%). The cost of VI per capita was £675(including wellbeing loss) or £180 (excluding wellbeing loss). These were equivalent to approximately 6.0% and 1.6% of the Gross National Income per capita (GNIpc = £11,212, $US18,380, GNI at Atlas Method), respectively. Monthly household income was reported as less than TT$9999 (£952, US$1,562) by 80% (*n* = 1985). This could indicate a bias toward lower self-reported income in our study, as average monthly household income of TT$9202 was reported in the 2008/2009 Household Budget Survey [34], or that the GNI represents skewed income distribution and substantial inequality; Trinidad and Tobago has reserves of oil and natural gas, and the last GINI coefficient estimate for Trinidad and Tobago was 40.2 in 1992 [35]. It is likely the economic burden of vision impairment fell disproportionately on lower income households, with 91% MSVI and 96% blind participants residing in households with monthly income <TT$9999, compared to 75% with normal vision. Visually impaired participants were significantly more likely to have lower educational attainment, and were less likely to have access to the internet or a family car.

The findings of this predominantly bottom-up, observational cost of vision loss study broadly agree with other studies in high-income countries. These similarly report that intangible effects, including estimates relating to loss of wellbeing, make the greatest contribution to the overall economic impact of VI and blindness [15]. After these, the next highest costs typically result from productivity losses, followed by caregiving, recurrent hospitalisations and use of medical and supportive devices, with drug costs not adding significantly in studies published before 2012 [15]. Direct comparisons of absolute costs are challenging because consensus on what to include in cost of vision loss studies was unavailable prior to 2010 [19], and there is still no standardisation of cost tools, with investigators frequently devising their own [15, 36]. In Trinidad and Tobago in 2014, direct expenditure on topical ophthalmic medications contributed significantly to total direct costs, and glaucoma was the leading cause of vision loss [16]. Given the large volumes of topical ophthalmic medications involved, there may be considerable benefit to the Ministry of Health intervening to reduce acquisition cost.

Costs in other domains were lower than expected for a high-income country, indicating a possible gap in eyecare service provision relative to the needs of the population. For example, no participant in 2014 had received laser refractive surgery, or anti-VEGF therapy (only available in the private sector) [18]. The latter was emerging as standard-of-care for numerous eye diseases internationally at that time. However, NESTT was not powered to reliably determine the prevalence of uncommon events. The relatively low unit cost of ophthalmology outpatient services may reflect historic underinvestment in public sector ophthalmic equipment. In 2014, only three of five public hospital eye departments in Trinidad and Tobago had visual field analysers, none had OCT imaging devices, and whilst two had FFA devices, they did not have sufficient staff for routine use [18]. We also observed few state-funded prevention programmes, with no national screening approach for congenital cataract or retinopathy of prematurity, responsible for some cases of adult VI. A pilot screening programme for diabetic retinopathy (DR) was completed in 2013, and a school vision screening programme was in the process of being implemented [18]. Low vision services were relatively underdeveloped for a high-income country, with only one low vision participant reporting previous assessment, and less than 10% reporting access to low vision aids. No blind people were employed, indicating potential opportunity to strengthen workplace-based enablement policies.

Societal direct costs are anticipated to have risen significantly in the decade following the 2014 national survey, through a combination of increased demand for eyecare services from an aging population, catch-up in ophthalmic management practices relative to other high-income countries driving a need for investment in equipment, and demand for higher-cost medicines and laser and surgical treatments.

Which disability weights are most applicable to VI remains controversial [27]. Our sensitivity analysis highlighted that disability weights from difference sources yield estimates of the cost associated with lost wellbeing which vary 4-fold, between 3485 DALYs (equivalent to TT$941,661,752) [26] and 12,259 DALYs (equivalent to TT$3,338,445,299) [7].

The study had a number of limitations. In common with previous studies, this study was limited to costs incurred by those aged 40 years and above [37–39]. Secondly, the survey did not include the institutionalised population, or consider costs associated with long-term care placement resulting from VI. Thirdly, the cost and utilisation estimates are subject to non-response bias (response rates 59–66%). Blind people may have been less likely to participate than normally sighted people, on account of transport difficulty, lack of someone to accompany them, frailty, or lack of perceived value to participating in the eye survey, and those blind people who attended clinic may have differed significantly from those who were housebound. This may account for our conservative estimate of informal care needs (23% blind people required a mean 8.4 h per day). A systematic review reported average informal care hours ranging from 5.8 h per week for persons with vision >20/32 to 94.1 h per week for persons with vision <20/250 [15]. Fourthly, the data were at risk of recall bias, with participants asked to recall episodes and costs occurring over the past 12 months. Some may have forgotten an entire episode of eyecare, or incorrectly recalled whether it occurred within or prior to the 12-month period in question [25, 40]. In common with many other cost studies, this analysis does not include transfer payments or estimation of dead-weight losses. This study was also unable to consider the opportunity cost of being a carer. Furthermore, direct health costs arising from falls, fractures, motor vehicle accidents, exacerbation of diabetic complications due to difficulty self-managing, and depression relating to VI were not explicitly measured in this survey. These have contributed substantially to direct costs in some studies [15]. Finally, it is important to highlight that whilst we estimated an assumed value of purchasing a DALY, and explored this in sensitivity analysis, as recommended by Frick et al. [19] cost-of-illness studies do not typically attach monetary values to the DALY or QALY impact of disease. We acknowledge that attempting to value statistical life – the amount that groups of individuals are willing to pay for a marginal change in their likelihood of death – is fraught with conceptual and ethical limitations.

Our study was subject to a number of assumptions. The market price for labour was assumed to be a reasonable approximation of the opportunity cost of the employment gap resulting from VI [25]. Furthermore, we assumed that market prices including an element of profit, such as private sector service and drug costs, were based on a fair rate of return on investment, such that they reflected societal opportunity costs [41].

These cost estimates provide an important benchmark and baseline data for cost-effectiveness analyses in Trinidad and Tobago. For example, to explore the potential value of widening access to low vision aids, workforce enablement programs, widening access to DR screening [42], widening access to affordable spectacles for effective refractive error coverage [43], or investment to improve effective cataract surgical coverage [44]. More generally, there is pressing need for international consensus on cost of VI study design and measurement tools, to facilitate comparison between countries [6, 15, 19, 45].

## Conclusion

This cost of vision loss study finds that VI in Trinidad and Tobago in 2014 had a significant societal economic impact, with affected individuals and their families bearing the majority of associated costs, including lost wellbeing, informal care costs, productivity losses and other opportunity costs. This highlights that policy decisions based on direct costs to the health sector alone would fail to apportion appropriate societal and research resources to addressing VI – at least 86% of which is potentially avoidable through interventions to prevent and treat eye disease [16]. We identified potential areas for investment, and elements that may drive rising future eyecare costs, including the aging demographic, emerging high-cost medicines and imaging equipment. Given the high proportion of indirect costs and intangible effects associated with VI, and Trinidad and Tobago’s pluralistic eye care system, policy makers may need to consider intervening proactively to correct market failures that limit timely access to sight-saving interventions in the public sector.

## Summary

### What is known about this topic?

- Understanding and mitigating the societal economic impacts of vision impairment (VI) will be critical to achieving the Sustainable Development Goals.
- Previous systematic reviews reveal considerable heterogeneity in the methodology and findings of cost-of-VI studies, with few based on individual-level cost and utilisation data from primary observational studies, including population-based eye surveys.
- Consensus Guidelines were proposed by Frick et al in 2010 to support greater standardisation, but leave some issues unaddressed.
- There were no data on the economic impact of vision impairment in the Caribbean.

### What this study adds

- We estimated the total societal cost of VI in 2014 at UK£365,650,241 (TT$3,842,324,655), equivalent to £675 per capita (in the population >40 years).
- Loss of wellbeing accounted for 73.3% of total cost, followed by indirect costs, direct medical costs and direct non-medical costs, highlighting the extent to which individuals and their families bear the societal economic cost of VI.
- This study provides detailed methodological outline showing how economic variables can be readily included in survey questionnaires, potentially enhancing the impact of population-based eye survey research.

### Supplementary information

Supplementary Tables

## Data Availability

Further analyses on the unpublished data set are in progress with manuscripts in preparation. Data access is governed by the NESTT Steering Committee, chaired by a representative of the Faculty of Medical Science at the University of the West Indies (St Augustine, Trinidad) and academic partners in the Vision and Eye Research Unit at Anglia Ruskin University (Cambridge, UK), and The School of Life Course and Population Sciences, King’s College London.
