# Supplementary material for: The societal economic impact of vision impairment in adults 40 years and above: findings from the National Eye Survey of Trinidad and Tobago
Source: Eye (Lond). 2023 Dec 8;38(11):2124–33. doi: 10.1038/s41433-023-02860-x (PMC11269728; doi:10.1038/s41433-023-02860-x)
Supplement: Supplementary file 1 — Supplementary Tables [file 41433_2023_2860_MOESM1_ESM.docx]

Supplementary Tables

Table 1: Content of the socioeconomic questionnaire developed for use in the National Eye Survey of Trinidad and Tobago and administered by a member of the survey team to participant face to face

| Question | Answer mode | Answer categories (if applicable) |
| --- | --- | --- |
| **EMPLOYMENT** | | |
| Did you have a job in the past 12 months? | Yes / No |  |
| Are you in paid employment at the moment? | Multiple choice (select one) | Yes - Full time,Yes - Part time, No |
| Which group best described your main occupation | Multiple choice (select one) | (examiner refers to ICO 2008 definitions of groups) Group 1 to 9 |
| Please detail **your** specific job | free text |  |
| What type of worker status applies to you? | Multiple choice (select one) | state owned enterprise / central or local government / government contract work /private establishment |
| Taking the past year, can you estimate the average income for your household (all adults) | numeric value |  |
| If you don’t know the amount, can you estimate the monthly household income? | Multiple choice (select one) | TT$ <1000 / 1000-4999 / 5000-9999 / 10,000-30,000 / >30,000 |
| How many days off sick have you had in the last 12 months? | Numeric value |  |
| Have you lost any earnings in the last 12 months as a result of visits to doctors or hospitals for eye/vision care | Yes / No |  |
| If yes, please estimate the amount of lost earnings | Numeric value |  |
| Have you lost any earnings in the last 12 months as a result of ill health relating to your eyes/vision | Yes / No |  |
| If yes, please estimate the amount of lost earnings | Numeric value |  |
| **HEALTH INSURANCE** | | |
| Which sector do you use for vision and medical care? | Multiple choice (select one) | Public / Private / Both |
| Are you covered by private health insurance or by a Group Health Insurance Plan at work? If no, no further questions in this section | Yes / No |  |
| If yes, which is your insurance company? | Multiple choice (select one) | Aligico/PanAmerican /Colonial Life / Guardian Life Maritime Life /Sagicor / TATIL/other |
| Other (please specify) | text value |  |
| What is your % co-insurance payment for the vision benefit? | % or don’t know |  |
| What is your maximum coverage under the vision benefit | numeric value |  |
| Who is the main policy holder? | Multiple choice (select one) | I am / My spouse or partner / My parent / My child / Other |
| **HEALTH SERVICE UTILISATION**  Over the past 12 months how many times have you: | | |
| Seen an eye doctor as an outpatient | numeric value |  |
| Seen an optometrist/ optician | numeric value |  |
| Seen a primary care doctor about your eyes/vision | numeric value |  |
| Visited a health centre about your eyes/vision | numeric value |  |
| Visited the emergency room about your eyes/vision | numeric value |  |
| Been admitted for day case eye surgery | numeric value |  |
| Spent a night in hospital on account of your eyes/vision | numeric value |  |
| Only answer if previous visits for eye care:  How do you normally travel from your home to eye/health care facilities? If you normally use more than one form of transport please indicate the way you most usually travel for the main (the longest in terms of distance) part of the journey | Multiple choice (select one) | private car / taxi / max-taxi / walk / bicycle / water taxi / housebound (not applicable) |
| **Out of pocket expenditure over the past 12 months**  Please estimate how much you or a family member have spent on the following for you: | | |
| Health insurance that covers eye care | numeric value |  |
| Eye drops | numeric value |  |
| Consultations with an optician/optometrist | numeric value |  |
| Consultations with an eye doctor | numeric value |  |
| Consultations with your primary care doctor relating to eye/vision problems | numeric value |  |
| Spectacles and/or contact lenses | numeric value |  |
| Laser treatment for your eyes | numeric value |  |
| Eye surgery | numeric value |  |
| Travel costs for all visits to clinics and hospitals relating to your eyes or vision | numeric value |  |
| Informal care for vision loss | | |
| Over the past year, how many hours per month have family or friends devoted to providing you with informal nursing or other care or transportation as a result of your eye or vision problems | numeric value |  |

Table 2: Direct medical care - Population-based estimation of the utilisation of different eye care services in the past year by people aged 40 years and above in Trinidad and Tobago in 2014

| Eye care provider | Crude prevalence % (n) | Adjusted prevalence  % (95% CI) | Adjusted mean episodes per person (95% CI) | Estimated number of people aged >40y using any care in 2014 | Estimated episodes of eye care in 2014 |
| --- | --- | --- | --- | --- | --- |
| Optometrist/ optician | 17.7 (444) | 16.8 (15.3- 18.4) | 1.02 (1.00-1.04) | 90,871 | 92,807 |
| Ophthalmol-ogist | 13.0 (328) | 12.1 (11.1- 13.2) | 1.52 (1.37-1.66) | 65,828 | 99,871 |
| General practitioner | 0.4 (10) | 0.33 (0.17- 0.62) | 1.36 (1.00-1.72) | 1,794 | 2,444 |
| Health centre | 0.5 (13) | 0.57 (0.36- 0.89) | 2.75 (2.03-3.48) | 3,065 | 8,440 |
| Emergency department | 0.2 (6) | 0.23 (0.11- 0.52) | 1.00 | 1,269 | 1,269 |
| Day case surgery | 0.4 (10) | 0.31 (0.17- 0.52) | 1.23 (0.99-1.47) | 1,678 | 2,062 |
| Overnight admission | 0.4 (11) | 0.42 (0.25- 0.68) | 3.15 (1.44-4.86) | 2,253 | 7,102 |
| TOTAL |  |  |  | 166,758 | 213,995 |

Table 3: Unit costs of direct medical eye care services and treatments in Trinidad and Tobago in 2013-2014 (TT$s 2014 (TT$1 = UK£0.0952) from a national contemporaneous eyecare system survey*

| Eye services and treatments | Mean Unit Cost TT$ | SD | Range | Provider responses  n (RR) |
| --- | --- | --- | --- | --- |
| PRIVATE SERVICES |  |  |  |  |
| Optometrist clinic | 113 | 27.8 | 60 to 200 | 48 |
| Ophthalmologist New clinic | 450 | 70.7 | 350 to 500 | 3 |
| Ophthalmologist Follow-up clinic | 283 | 23.6 | 250 to 300 | 3 |
| GP (Private) clinic | 283 | 40 | 265 to 300 | 2 |
| A&E (Private hospital) visit | 500 | NA | NA | 1 |
| PUBLIC SERVICES |  |  |  |  |
| Health Centre | 373 | NA | NA | 1 |
| Accident and Emergency | 1100 | NA | NA | 1 |
| Eye Out-patient clinic | 700 | NA | NA | 1 |
| TREATMENTS |  |  |  |  |
| Basic spectacles | 806 | 313 | 400 to 1500 | 48 |
| Bi/tri/varifocal spectacles | 1370 | 555 | 575 to 2500 | 48 |
| Cataract with intraocular lens | 11,333 | 943 | 2500 to 4000 | 1 |
| Day case admission | 1200 | NA | NA | 1 |
| Overnight admission | 2000 | NA | NA | 1 |
| Anterior segment laser | 1683 | 131 | 1500 to 1800 | 3 |
| Glaucoma laser | 2250 | 250 | 2000 to 2500 | 3 |
| Macula/retina laser | 3167 | 624 | 2500 to 4000 | 3 |
| Eye drops public (CDAP) | 50 | 30 | 19 to 84 | 1 |
| Eye drops private | 101 | 30 | 67 to 144 | 3 |

KEY: CDAP Chronic Disease Assistance Plan; n number of survey responders; RR survey response rate; NA not available

* Braithwaite T, Winford B, Bailey H, et al. Health system dynamics analysis of eyecare services in Trinidad and Tobago and progress towards Vision 2020 Goals. *Health policy and planning.* 2018;33(1):70-84.

Table 4: Direct medical care: Prescription eye drop use, compliance and costs, extrapolated to the national population aged >40 years in Trinidad and Tobago in 2014 (TT$s 2014 (TT$1 = UK£0.0952))

| Variables | Results and explanatory notes |
| --- | --- |
| Eye drop utilisation in the past 3 months, by person | Of 2792 NESTT participants, 186 reported right eye drop use (adjusted population-based prevalence of eye drop usage in adults >40 years was 5.4%, 95 % CI 4.8-6.2)), and 181 reported left eye drop use (adjusted prevalence 5.4 % (95% CI 4.7-6.1)). |
| Mean eye drop prescriptions per eye per month | In total, 367 eyes were receiving 509 prescribed eye drops, and the adjusted mean eye drop prescriptions per eye per month amongst those prescribed any was 1.7 (95 % CI 1.6-1.8) in the right eye and 1.6 (95 % CI 1.5-1.7) in the left eye. Thus, in the population aged 40 years and above in 2014, an estimated 58,516 eyes were prescribed 1,152,803 bottles of eye drops. |
| Non-compliance with prescribed eye drops | Participants were asked if they ever forget to insert their eye drops, and 86 (46.2%) reported that they forget. The frequency of non-compliance was reported by 76 of these participants to be: less than once per month (n=17, 22.4 %), several times per month (n=21, 27.6 %), several times per week (n=15, 19.7 %), most days (n=5, 6.6 %), and 18 (23.7 %) participants reported that they are not using the prescribed eye drops at all. |
| Reasons for non-compliance with prescribed eye drops | The reasons for non-compliance (given by 75 participants) included forgetting (n=49, 65.3 %), running out and not being able to afford to buy more (n=8, 10.7 %), running out and having difficulties getting more on account of transport issues (n=8, 10.8 %), believing that the eye drops were not effective (n=6, 8.0 %), running out and finding it difficult to get more on account of stock issues (n=3, 4.0 %), and disliking the drops on account of side effects (n=1, 1.3 %). |
| Not included in this study | These estimates did not include over-the-counter eye drops (e.g. ocular lubricants), and were therefore a conservative estimate of total eye drop utilisation by the national population in this age group. |
| Cost of and access to prescription eye drops in 2014 | In 2014, four eye drops for the management of ocular hypertension and glaucoma (pilocarpine 2 or 4%, betaxolol hydrochloride, and timolol maleate) were available at no cost to the patient in the public sector, via the Chronic Disease Assistance Plan (CDAP). Other agents available via CDAP included antimicrobial agents moxifloxacin and oxytetracycline/polymixin B, and olopatadine hydrochloride for allergy. All other eye drops required over-the-counter purchase, or private prescription.  The average cost of four intraocular pressure lowering agents was TT$50.34 from the government supplier. The average cost of the same agents in the private sector was TT$100.52. The average unit cost to the health system of other commonly prescribed drugs (anti-inflammatory, anti-allergy, and anti-microbials) was TT$49.39 from the government supplier, and TT$111.09 from private pharmaceutical companies. The estimates of the cost to the patient of the latter were conservative, because it was likely that individual retail pharmacies added profit margin to the cost price of the drug.  The estimated total cost of prescription eye drops, of TT$87,303,053, was reduced by 23.7% to account for the percentage of non-compliance reported, to TT$66,612,229, and explored in sensitivity analysis (ranging from 52.6 to 100% compliance). |
| Clinical indication for eye drops | The majority of prescribed eye drops were for the management of glaucoma, the leading cause of blindness in Trinidad and Tobago in 2014. Some people were receiving four different pharmacological agents. The cost of glaucoma drops was therefore used for cost estimation in this analysis. |

Table 5: Direct medical care: Utilisation, indication for and cost of laser eye therapy in the population aged >40 years in Trinidad and Tobago 2014 (TT$s 2014 (TT$1 = UK£0.0952))

| Variables | Results and explanatory notes |
| --- | --- |
| Utilisation of eye laser in the past 12 months (private sector) | Laser therapy in the private sector was reported by 0.2% (n=6/2792) NESTT participants. Extrapolating to the national population, an estimated 1165 people received laser in the private sector in 2014. |
| Utilisation of eye laser in the past 12 months (public sector) | The number of laser procedures performed in the past 12 months in the public sector was not ascertained from NESTT participants directly. However, the concurrent eye care system study* found that 1433 laser procedures were performed in four out of five of the public hospitals in 2013-2014, serving 81.6% of the national population. Two assumptions, namely that all laser-treated patients were 40 years and above, and that each person had only one laser procedure, yields an estimate that 0.32% of the population served by these four hospitals had received laser treatment. Extrapolating to the national population, an estimated 1756 people received laser in the public sector in 2014. |
| Indications for laser | Previous laser at any time in the past was reported for 82 right eyes and 78 left eyes (2.9%). The indications included diabetic retinopathy (88 eyes), retinal tears (13 eyes), posterior capsule opacification following cataract surgery (33 eyes), and glaucoma (21 eyes). No one aged 40 years and over reported laser refractive correction, and the remainder of laser indications were not specified, generally because of recall difficulty. |
| COST OF LASER | Unit costs for 2014 were applied assuming that 65.2% lasers were to the retina, 21.3% were to the anterior segment (e.g. laser capsulotomy), and 13.5% were for anterior segment glaucoma treatment (e.g. peripheral iridotomy). Total cost was TT$7,963,698. |

* Braithwaite T, Winford B, Bailey H, et al. Health system dynamics analysis of eyecare services in Trinidad and Tobago and progress towards Vision 2020 Goals. *Health policy and planning.* 2018;33(1):70-84.

##

**Table 6: Direct medical care: Utilisation, indication for and cost of other eye care treatments in the population aged >40 years in Trinidad and Tobago in 2014**

| Treatments | Results and explanatory notes |
| --- | --- |
| Intravitreal injection of anti-vascular endothelial growth factor (VEGF) | This therapy was not available in the public health sector in 2014 for any indication (including retinal vein occlusion, diabetic macular oedema or wet age-related macular degeneration). No NESTT participants reported receiving these in the private sector. |
| Ophthalmic surgery other than cataract extraction | More specialised ophthalmic surgery than cataract extraction was also not available in the public sector (e.g. vitreoretinal surgery, corneal transplant surgery, oculoplastic surgery) in 2014. |
| Oral medication for eye disease | No participants reported use of oral medications for the treatment of eye disease in the past 3 months. |

Table 7: The crude proportion of NESTT participants reporting any out of pocket expenditure (OOPE) on eye care services, refractive correction and eye drops over the past 12 months, and the median OOPE in each category amongst those reporting any (TT$s 2014 (TT$1 = UK£0.0952))

| Eye care expenditure | Crude %(n) with any OOPE | Crude OOPE (TT$) amongst those with any OOPE, median (IQR), range |
| --- | --- | --- |
| Optometry review | 16.0 (402) | 100 (100 to 150), 50 to 2500 |
| Ophthalmology review | 6.7 (170) | 400 (300 to 550), 100 to 12000 |
| GP review for eyes/vision | 0.1 (3) | 400 (100 to 2000), 100 to 2000 |
| Day case surgery for eyes/vision | 0.7 (18) | 10000 (1600 to 13000), 200 to 100000 |
| Laser therapy (excluding refractive) | 0.2 (6) | 4500 (3000 to 5000), 1000 to 10000 |
| Laser refractive therapy | 0 | 0 |
| Any eye care services | 20.5 (513) | 150 (100 to 350), 50 to 101500 |
| Refractive correction | 14.0 (353) | 2400 (1700 to 3000), 20 to 10000 |
| Eye drops (any) | 4.3 (108) | 800 (220 to 2450), 35 to 12000 |
| Anti-VEGF intravitreal injection therapy | 0 | 0 |

KEY: IQR Interquartile range; OOPE Out of pocket expenditure; TT Trinidad & Tobago

Table 8: Direct non-medical: Utilisation and total cost for refractive correction in the population aged >40 years in Trinidad and Tobago in 2014

| Refractive correction variable | Crude prevalence  % (n) | Adjusted population-based prevalence % (n) | Estimated people affected in national population >40 years in 2014 |
| --- | --- | --- | --- |
| Utilisation of spectacles or contact lenses in the past 12 months | 14.0 (353/2524) | 13.3 (95% CI 11.9-14.8) | 72,194 |
| Cost assumptions | We assumed that 70% of spectacles purchased were basic distance or near spectacles, and 30% were bi, tri or varifocal spectacles. | | |
| Total cost | The total annual expenditure on spectacles was estimated to be TT$70,391,870, or £6,698,758, equivalent to £12.36 per capita. | | |

Table 9: Direct non-medical: Low vision device usage and support service access reported by vision impaired (<6/18 in better-seeing eye) people aged >40 years in Trinidad and Tobago in 2014

| Low vision variable | Crude prevalence in NESTT sample  % (n) | Adjusted population-based prevalence  % (n) | Number affected in national population aged >40 years in 2014 |
| --- | --- | --- | --- |
| Best-corrected vision impairment (<6/18) in the better seeing eye | 3.4% (n=120/3578) | 3.05% (95% CI 2.56-3.63) | 16,535 (95% CI 13,882-19,675) |
| Previous low vision assessment amongst the vision impaired | The NESTT low vision questionnaire was completed by 80.8%(n=97/120) participants with best-corrected vision impairment worse than 6/18 in the better seeing eye. This group ranged in age from 41 to 99 years (mean age 74.1, sd 12.8) and 51.6%(sd 50) were female. Only one person had received a low vision assessment previously. | | |
| Desire for low vision assessment | Participants were asked if they felt they would benefit from a low vision assessment: 26.0%(n=25) replied “yes”; 45%(n=44) were “unsure”, and 29%(n=28) replied “no” | | |
| Barriers to low vision assessment | Barriers to having a low vision assessment were reported by some participants to include lack of access to this service (n=11), transport problems (n=2), cost of low vision aids (n=1), and the lack of availability of low vision aids in Trinidad and Tobago (n=2). | | |
| Utilisation of low vision aids | Use of the following low vision aids was reported: 9.6% (n=10) used a white stick or cane; 3.9% (n=4) used a visor; 6.7% (n=7) used tints or shields; 1% (n=1) used a typoscope; 1% (n=1) used closed circuit television; 1.0% (n=1) used audio or DVDs, 3.9% (n=4) used talking electronic devices; 1.0% (n=1) used Braille; 1.0% (n=1) used a hand held magnifier; 1.0% (n=1) used a stand magnifier; 2.9% (n=3) used spectacle magnification; 2.9% (n=3) used magnification software; 1.9% (n=2) used speech software; and 1.0% (n=1) used a braille keyboard. No visually impaired participants reported use of a guide dog, GPS, liquid level indicators, mounted telescopes, speech input software, or contrast enhancing software. | | |
| Availability and cost of low vision assessment clinics in 2014 | In the contemporaneous eye care system study*, 35.4% (n=17/48) of responding optometrists reported offering low vision services; 7 reported offering assessment at no cost to clients (via the University of the West Indies Department of Optometry Low Vision Clinic), and the remaining 10 offered assessment for a mean fee of TT$196 (sd 93.95, range 60 to 400) | | |
| Total cost of low vision aid devices and assessment | TT$37,576,201 | | |
| Excluded from this study | We did not ask NESTT survey participants about home or workplace adaptations such as stair lifts, door-opening devices, and ramps.  The unit price of individual low vision aids in Trinidad and Tobago in 2014 was not determined. We took costs from previously published sources including a study on the cost of low vision aids in four European countries in 2004, [^16^](#_ENREF_16) and the Royal National Institute for the Blind United Kingdom online shop.[^19^](#_ENREF_19) These costs were adjusted to 2014 values. | | |

* Braithwaite T, Winford B, Bailey H, et al. Health system dynamics analysis of eyecare services in Trinidad and Tobago and progress towards Vision 2020 Goals. *Health policy and planning.* 2018;33(1):70-84.

Table 10: Direct non-medical: Cost and frequency of transportation to and from eyecare services for the population aged >40 years in Trinidad and Tobago in 2014 (TT$s 2014 (TT$1 = UK£0.0952))

| Transportation variable | Results and explanatory notes |
| --- | --- |
| Preferred main transportation mode to attend eye care services | Private car (58.8 %, n = 379/645), maxi-taxi or bus (21.6 %, n = 139/645), taxi (17.7 %, n = 114/645), walking (1.1 %, n = 7/645) and taking the water taxi (0.9 %, n = 6/645). |
| Unit cost of a return journey in 2014 | Mean costs were determined in the health system survey*, in which 450 public hospital patients attending out-patient clinics in all 5 regions of Trinidad and Tobago were surveyed to establish unit costs: TT$20 for a maxi, TT$60 for a private car, TT$80 for a taxi, and TT$80 for a water taxi. These unit costs assumed attendance at an eye care facility located within the same region as the home address. |
| Mean travel cost for a return journey | Applying the proportions reporting different modes of transport and the unit cost for each, the mean travel cost was TT$54.48 for each return journey. |
| Total episodes of eye care in 2014 | We summed visits to optometrists, ophthalmologists, , health centres, emergency departments, day case admissions and overnight admissions, (with the latter divided by the adjusted mean length of each admission (3.15 days)) to estimate 209,145 episodes of eye care in 2014 |
| Total estimated expenditure on transport | We multiplied total episodes of eye care by mean travel cost for a return journey to estimate a total transport expenditure in 2014 of TT$11,394,220. |
| Not included in this study | Travel costs incurred by family or friends accompanying an individual to their eye care service appointments were not included, so this estimate was conservative. |

*Braithwaite T, Winford B, Bailey H, et al. Health system dynamics analysis of eyecare services in Trinidad and Tobago and progress towards Vision 2020 Goals. *Health policy and planning.* 2018;33(1):70-84.

**Table 11: Indirect costs a) Productivity loss in the 40 to 64-year-old age group in 2014, by vision category, estimated indirectly from the employment rate gap resulting from vision impairment (TT$s 2014 (TT$1 = UK£0.0952))**

| Vision category | n | ER* | Gap | Median annual income TT$ | Individual productivity loss TT$ | Cases in 2014 | TOTAL Productivity Loss  TT$ |
| --- | --- | --- | --- | --- | --- | --- | --- |
| Normal | 1561 | 73.2 | -0.07 | 54000 | -2638.8 | 258726 | -682735706.6 |
| Near VI | 795 | 63.9 | 0.06 | 54000 | 2366.9 | 134244 | 317737113.8 |
| Mild VI | 94 | 54.5 | 0.20 | 54000 | 7432.2 | 16431 | 122120496.6 |
| MSVI | 69 | 41.4 | 0.39 | 54000 | 14505.9 | 11103 | 161064270.2 |
| Blind | 5 | 0.0 | 1.00 | 54000 | 36868.7 | 812 | 29937439.33 |
| TOTAL | 2524 | 68.3 |  |  |  | 421316 | 630,859,319.9 |

ER* Adjusted employment rate; MSVI moderate and severe vision impairment

**Table 12: Indirect costs b) Productivity loss associated with part-time work (assuming 50% working hours) (TT$s 2014 (TT$1 = UK£0.0952))**

| Vision category | % Part Time  work | ER for PT | Gap PT | Median annual income TT$ | Productivity loss | Estimated part time workers n | Additional productivity loss for part time work |
| --- | --- | --- | --- | --- | --- | --- | --- |
| Normal | 11.1 | 69.1 | -0.08 | 54000 | -2936.9 | 258,726 | -77,107,869.5 |
| Near VI | 15.2 | 59.0 | 0.08 | 54000 | 2864.2 | 134,244 | 66,757,509.0 |
| Mild VI | 17.9 | 49.6 | 0.22 | 54000 | 8292.6 | 16,431 | 14,137,453.1 |
| MSVI | 12.0 | 38.9 | 0.39 | 54000 | 14450.2 | 11,103 | -618,734.4 |
| Blind | 0.0 | 0.0 | 1.00 | 54000 | 36868.7 | 812 | 0.0 |
| TOTAL | 12.5 | 64.0 |  |  |  |  | 80,276,227.7 |

KEY: ER employment rate; MSVI moderate and severe vision impairment; PL productivity loss; PT part time (assumed to be 50% working hours); TT Trinidad and Tobago; VI vision impairment.

**Table 13: Productivity loss associated with sick leave in the population aged 40 to 64 years who reported employment in the past 12 months (TT$s 2014 (TT$1 = UK£0.0952))**

| **Variable** | **Crude data from NESTT Participants** | **Responders n** | **Total population-based estimate**** |
| --- | --- | --- | --- |
| **NESTT participants who reported taking 1 or more days of sick leave in the past 12 months** | 37.2% (438/1178)* | 1178 |  |
| **Number of sick days taken amongst those reporting any** | Mean 7.9 (sd 20.6) days  Median 4 (IQR 3-7), ranging from 1 to 365 days | 438 |  |
| **NESTT participants reporting any lost earnings specifically on account of healthcare visits for eye or vision care in past 12 months** | 0.9 % (11/1248) | 1248 | 2535 people |
| **Estimate of lost income associated with vision/eye health care in past 12 months, median (IQR), range** | TT$ 2450 (700-8938), range 300-25000 | 8 | TT$6,211,776 (IQR 1,774,793-22,661,571). |
| **NESTT participants reporting missing work days on account of poor eye health/poor vision without loss of income** | 1.5% (18/1239) | 1239 |  |

*This indicates a high rate of absenteeism in the population

**Assuming 421,316 people aged 40-64 in the natioanl population mid-2014.

Table 14: Productivity loss associated with informal care for blind individuals

| Utilisation or cost item | Value, units, range if applicable |
| --- | --- |
| Reported use of any informal care in past 12 months amongst blind participants attending the regional NESTT clinic* | 23.1 % (n=3/13) |
| Hours of informal care amongst those reporting any, in a typical month, mean (sd), 95% CI, range | 260 (209) hours, 23-497, 20-400 |
| Unit cost of an hour of informal care | TT$ 21.2 |
| Estimated number of blind people aged 40 years and above in Trinidad and Tobago in 2014 | 3956 (95% CI 2601- 5256) blind people |
| Estimated number of blind people requiring informal care | 914 blind people needing informal care |
| TOTAL COST OF INFORMAL CARE FOR BLIND PEOPLE (assuming 23.1% utilising) | TT$ 5,032,032 |

*Those with Near VI, mild VI, and MSVI reported less than one hour of care per month.

## **Table 15:** **Estimation of Disability Adjusted Life Year loss in 2014**

| Vision category | 2014 cases | DW | pYLD | YLL | DALY | 95 % CI | 95 % CI |
| --- | --- | --- | --- | --- | --- | --- | --- |
| Mild VI | 31,267 | 0.005 | 156 | 0 | 156 | 137 | 176 |
| Moderate VI | 26,661 | 0.089 | 2373 | 0 | 2373 | 2055 | 2686 |
| Severe VI | 2,547 | 0.314 | 800 | 0 | 800 | 476 | 1106 |
| Blind | 3,956 | 0.338 | 1337 | 0 | 1337 | 879 | 1777 |
| Near VI | 120,842 | 0.047 | 5680 | 0 | 5680 | 5298 | 6062 |
| TOTAL vision loss | 185,273 |  | 10346 |  | 10346 | 8845 | 11806 |

KEY: pYLD prevalent years lived with disability; YLL Years of life lost; DALY disability adjusted life years; VI vision impairment; DW disability weight

Table 16: Estimation of transfer payments (excluded from total cost estimate)

| Transfer payments | Notes and cost estimation |
| --- | --- |
| Estimated annual cost of the disability allowance awarded to blind people of working age | Out of 3416 people aged 40 years and above, 1.7 % (58) reported disabled status, with an adjusted prevalence of 1.7 % (95 % CI 1.2-2.1). This varied by severity of presenting VI, from 0.9 % (95 % CI 0.4-1.5) in those with normal vision, to 27.3 % in those who were blind (95 % CI 23.8-30.8). Out of an estimated 3956 people who were blind in Trinidad and Tobago, 363 were aged 40 to 59 years (prevalence estimates applied to census data in each 5-year age group)*. The estimated annual cost of the disability allowance awarded to 27.3% (n=99) of these individuals was TT$1,784,958 (£169,863). |
| Blind Welfare Association budget in 2013/14 | The 2013/2014 budget of the Blind Welfare Association was TT$10,800,000, with the Government providing $7,922,000 and the remaining balance received from fundraising campaigns (sales, private contributions, donations), and rental income (Government of the Republic of Trinidad and Tobago, 2015). This non-profit, voluntary rehabilitation organization provided services for the blind, and reported serving about 1400 clients (Parliament of the Republic of Trinidad and Tobago, 2014). |
| Persons Associated with the Visually Impaired (PAVI) budget in 2013/2014 | The 2013/2014 budget allocated to Persons Associated with the Visually Impaired (PAVI), was $520,500 in 2014 (Government of the Republic of Trinidad and Tobago, 2015). |

*Braithwaite T, Verlander NQ, Peto T, et al. National Eye Survey of Trinidad and Tobago (NESTT): prevalence, causes and risk factors for presenting vision impairment in adults over 40 years. *The British journal of ophthalmology.* 2020;104(1):74-80.

Table 17: INDIRECT COSTS: Category of household monthly income by level of presenting vision (TT$s 2014 (TT$1 = UK£0.0952))

| Household monthly income | Normal vision  % (n) | NVI, normal distance | Mild VI  % (n) | MSVI  % (n) | Blind  % (n) |
| --- | --- | --- | --- | --- | --- |
| <TT$1000 | 0.6 (9) | 0.9 (7) | 1.0 (2) | 0.5 (1) | 0 |
| TT$1000-4999 | 30.8 (454) | 46.8 (360) | 48.7 (96) | 52.7 (98) | 55.6(15) |
| TT$5000-9999 | 43.3 (637) | 38.4 (295) | 41.6 (82) | 40.3 (75) | 40.7 (11) |
| TT$10,000-30,000 | 22.7 (334) | 13.4 (103) | 8.1 (16) | 6.5 (12) | 3.7 (1) |
| TT$ >30,000 | 2.7 (39) | 0.5 (4) | 0.5 (1) | 0 | 0 |
| Total | 100.0 (1473) | 100.0 (769) | 100.0 (197) | 100.0 (186) | 100.0 (27) |

## **Table 18: Information Commissioner’s Office (ICO) employment group by category of presenting vision**

| ICO employment group | Normal | Near VI | Mild VI | MSVI | Blind | Total |
| --- | --- | --- | --- | --- | --- | --- |
| Professionals | 43.6 (331) | 26.5 (85) | 10.8 (4) | 38.5 (10) | 0 | 37.6 (430) |
| Elementary occupation | 18.4 (140) | 24.3 (78) | 32.4 (12) | 23.1 (6) | 0 | 20.6 (236) |
| Service workers | 16.2 (123) | 20.9 (67) | 35.1 (13) | 19.2 (5) | 0 | 18.2 (208) |
| Clerical support work | 5.4 (41) | 2.2 (7) | 0 | 0 | 0 | 4.2 (48) |
| Technicians | 4.3 (33) | 2.8 (9) | 2.7 (1) | 3.9 (1) | 0 | 3.9 (44) |
| Machine operators | 4.0 (30) | 7.8 (25) | 5.4 (2) | 0 | 0 | 5.0 (57) |
| Craft | 3.0 (23) | 6.9 (22) | 2.7 (1) | 7.7 (2) | 0 | 4.2 (48) |
| Armed forces | 2.9 (22) | 2.8 (9) | 2.7 (1) | 0 | 0 | 2.8 (32) |
| Skilled agricultural | 2.1 (16) | 5.9 (19) | 8.1 (3) | 7.7 (2) | 0 | 3.5 (40) |
| Total | 100 (759) | 100 (321) | 100 (37) | 100 (26) | 0 | 100 (1143) |

Table 19 Highest education level completed by category of presenting vision

| Highest education completed | Normal vision  % (n) | Near VI  % (n) | Mild VI  % (n) | MSVI  % (n) | Blind  % (n) | Total  % (n) |
| --- | --- | --- | --- | --- | --- | --- |
| Primary | 37.4 (538) | 57.4 (413) | 69.6 (112) | 76.9 (120) | 84.6 (11) | 48.0 (1194) |
| Secondary | 40.6 (584) | 36.2 (260) | 22.4 (36) | 16.7 (26) | 7.7 (1) | 36.5 (907) |
| Post-secondary | 12.7 (182) | 3.1  (22) | 6.2 (10) | 2.6 (4) | 7.7 (1) | 8.8 (219) |
| University | 9.3 (134) | 3.3 (24) | 1.9 (3) | 3.9 (6) | 0 | 6.7 (167) |
| Total | 100 (1438) | 100 (719) | 100 (161) | 100 (156) | 100 (13) | 100 (2487) |

**Table** **20: Eye care episodes, health insurance status and preference for exclusive public sector use for eye care/vision, by category of vision**

| Prevalence | | Vision category | |  |  |  |
| --- | --- | --- | --- | --- | --- | --- |
|  |  | **Normal** | **Near VI** | **Mild VI** | **MSVI** | **Blind** |
| Any eye care episodes in past 12 months | **Crude %**  **(n/N)** | 32.2  (468/1452) | 18.5(135/728) | 29.2(47/161) | 31.7(50/158) | 7.7(1/13) |
|  | **Adjusted %,95% CI** | 30.7  (28.4-33.2) | 18.2  (15.9-20.7) | 26.4(20.6-33.2) | 32.1(25.3-39.8) | 4.6(0.7-24.0) |
|  | **OR***  **95% CI** | 1.0 | 0.5(0.4-0.6) | 0.7(0.5-1.1) | 0.75(0.5-1.1) | 0.1(0.0-0.9) |
| Health insured | **Crude %**  **(n/N)** | 24.0 (473/1968) | 10.7  (113/1053) | 9.6(21/218) | 3.8(8/212) | 0(0/30) |
|  | **Adjusted %,95% CI** | 24.6  (21.8 to 27.6) | 12.0  (10.2 to 14.2) | 9.9(6.5 to 14.9) | 5.1(2.6 to 9.9) | 0 |
|  | **OR***  **95% CI** | 1.0 | 0.4(0.3-0.5) | 0.7(0.4-1.1) | 0.3(0.1-0.5) | 0 |
| Public sector use only | **Crude %**  **(n/N)** | 11.2 (165/1470) | 29.0(218/751) | 30.3(54/178) | 47.2(85/180) | 54.2(13/24) |
|  | **Adjusted %,95% CI** | 10.46  (8.8-12.3) | 27.0  (23.9-30.3) | 30.7(24.1-38.2) | 41.9(35.0-49.1) | 60.0(45.1-73.0) |
|  | **OR***  **95% CI** | 1.0 | 3.3(2.6-4.2) | 2.7(1.9-4.0) | 5.7(3.9-8.2) | 7.1(2.9-17.4) |

* Prevalence estimate adjusted for multilevel design (island, cluster), weighted for response rate (by cluster), with post-stratification adjustment to 2011 Census population stratified by municipality (15), 5-year age groups and gender

** Odds of outcome, by vision category, adjusted for age and sex in multivariable model. Global Wald p< 0.001

Table 21: Percentage of population sample reporting out of pocket expenditure (OOPE) on various sources of direct medical costs, and amount of expenditure in TT$, by presenting vision level

| Outcome variable | Normal vision | Near VI | Mild VI | MSVI | Blind |
| --- | --- | --- | --- | --- | --- |
| Reporting any OOPE on eye care % (95% CI) | 25.3  (22.9-27.8) | 10.5  (8.8-12.4) | 15.1  (10.8-20.9) | 12.7  (8.2-18.9) | 0 |
| Amount of annual OOPE, all  Mean (95% CI), TT$ | 117  (74.4-159.8) | 228  (41.5-414.4) | 262.6  (-11.7-537) | 79.5  (6.7-152.3) | 0 |
| Amount of annual OOPE, in subgroup reporting any,  Mean (95% CI), TT$ | 511.2  (406.1-616.4) | 2540.0  (2066.0-3014.0) | 2589.0  (45.2-5132.9) | 998.3 (449.7-1547.0) | 0 |
| Reporting OOPE on refractive correction  % (95% CI) | 20.3  (18.1-22.7) | 4.9  (3.7-6.5) | 4.0  (2.1-7.6) | 1.3  (0.3-5.1) | 0 |
| Amount of OOPE on refractive correction, all  Mean (95% CI), TT$ | 492.9  (429.6-556.1) | 117.4  (73.9-160.8) | 76.4  (22.8-130.1) | 38.6  (-23.5-100.7) | 0 |
| Amount of OOPE on refractive correction, if any  Mean (95% CI), TT$ | 2405.5  (2321.3-2489.7) | 2472.2 (2131.4- 2813.0) | 1964.5 (1664.2-2264.7) | 2935.0 (1965.3-3904.7) | 0 |
| Reporting OOPE on eye drops  % (95% CI) | 4.7  (3.7-6.0) | 2.7  (1.8-4.0) | 5.4  (2.9-10.0) | 4.6  (2.2-8.9) | 19.3  (9.9-34.1) |
| Amount of OOPE on eye drops, all  Mean (95% CI), TT$ | 77.4 (43.8- 111.0) | 57.2 (9.5- 104.9) | 57.7 (17.8- 97.6) | 190.0 (-46.8- 426.8) | 808.8 (419.5-1198.0) |
| Amount of OOPE on eye drops, in subgroup with any  Mean (95% CI), TT$ | 1462.4  (1348.6-1576.2) | 2967.1 (2482.7-3451.4) | 1068.8  (586.5-1551.1) | 3142.0 (1560.4-4723.5) | 2754.3 (1800.1-3708.5) |

KEY: VI vision impairment
